# FoCup, a secreted protein, is essential for virulence of *Fusarium oxysporum* f. sp. *cucumerinum* on cucumber

**DOI:** 10.3389/fmicb.2025.1728884

**Published:** 2025-12-17

**Authors:** Ji-Tong Xu, He Liu, Shi-Dong Li, Rong-Jun Guo, Man-Hong Sun, Chao-Ge Yu, Zhou-Ping Sun, Xiao-Hong Lu

**Affiliations:** 1 College of Horticulture, Shenyang Agricultural University, Shenyang, Liaoning, China; 2 State Key Laboratory for Biology of Plant Diseases and Insect Pests, Institute of Plant Protection, The Chinese Academy of Agricultural Sciences, Beijing, China

**Keywords:** *Fusarium oxysporum* f. sp. *cucumerinum*, cucumber, virulence, effector, cupredoxin domain

## Abstract

*Fusarium oxysporum* f. sp. *cucumerinum* (*Foc*) infected cucumber (*Cucumis sativus*), leading to serious wilt disease and great economical losses worldwide. During infection, *Foc* secreted various protein effectors to facilitate colonization and disease development. Here, we identified a novel virulence effector, designated FoCup, which was highly up-regulated during *Foc-*cucumber interactions according to transcriptomic data. Bioinformatic analysis using SignalP-5.0 and InterPro predicted an N-terminal signal peptide and a cupredoxin domain in FoCup. Phylogenetic analysis indicated that FoCup is highly conserved within the *Fusarium* genus. Its secretory capability was experimentally confirmed by the yeast invertase secretion assay. Subcellular localization in *Nicotiana benthamiana* leaf cells revealed that FoCup-GFP predominantly localized to the plasma membrane, co-localizing with the membrane marker CD3-1007 (AtPIP2A-mCherry). Functional characterization demonstrated that Δ*FoCup* knockout mutants exhibited significantly reduced virulence on cucumber, accompanied by decreased conidiation, and increased sensitivity to osmotic stressors (e.g., glycerol, sorbitol, NaCl, and KCl). In contrast, mycelial growth remained comparable to the wild-type (WT) strain. The impaired virulence and conidiation in the knockout mutants (Δ*FoCup*) were fully restored in the complementary mutants (Δ*FoCup+FoCup*). Specifically, pathogenicity tests showed that the disease index caused by Δ*FoCup* was significantly reduced by 54.5and 62.5 compared to the wild type *Foc*, underscoring the critical role of FoCup in pathogenesis. Our findings provide new insights into the molecular mechanisms underlying *Foc* virulence.

## Introduction

Cucumber (*Cucumis sativus*), a major vegetable crop cultivated on over 1.2 million ha in China, is commonly suffering from *Fusarium* wilt disease. This soilborne pathogen causes characteristic symptoms including wilting, leaf yellowing and vascular discoloration (Cerkauskas et al., 2001). With an annual incidence rate of 10–30%, the disease results in substantial yield losses (Shen et al., 2017). Its causal agent, *Fusarium oxysporum* f. sp. *cucumerinum* (*Foc*), is one of the 143 documented formae speciales of *F. oxysporum*, and exhibits host specificity to cucumber (Edel-Hermann and Lecomte, 2019).

Conventional management strategies for cucumber *Fusarium* wilt, including agricultural practices and chemical applications, often exhibit limited efficacy due to the pathogen’s ability to produce long-lived chlamydospores and persist in soil and plant debris (Fravel et al., 2003; Hajji-Hedfi et al., 2018; Edel-Hermann and Lecomte, 2019). Furthermore, the complex genetic basis of host resistance poses substantial challenges in breeding stable, disease-resistant cultivars (Jaswal et al., 2020). A comprehensive understanding of *Foc* pathogenic mechanisms is therefore critical for developing innovative and sustainable control measures.

Secreted proteins of *F. oxysporum* have been extensively demonstrated to play pivotal roles in host plant infection. These virulence factors can be categorized into three major functional groups. Cell wall-degrading enzymes (CWDEs), particularly polygalacturonases (PGs), which hydrolyze structural polysaccharides like cellulose and pectin (King et al., 2011). In *F. oxysporum* f. sp. *lycopersici* (*Fol*), deletion of *pg1* and *pgx6* – two of eight PG-encoding genes significantly reduced both secreted PG activity and virulence, suggesting synergistic action of exo- and endo-PGs during pathogenesis (Bravo Ruiz et al., 2016). To facilitate infection and suppress host defense response, *F. oxysporum* also secretes mycotoxins, such as fusaric acid, fumonisin, beauvericin, enniatin and trichothecenes, etc. (Bani et al., 2014; Singh et al., 2017). Most importantly, a group of cysteine-rich effector proteins, particularly the SIX (secreted in xylem) proteins, have been identified from *Fol* infected tomato xylem sap (Armstrong et al., 1978). Genetic evidence demonstrated that specific SIX effectors (SIX1, SIX3, and SIX4) interact with corresponding tomato resistance proteins (I-1, I-2, and I-3), playing dual roles in both pathogen virulence and activation of host immunity (Widinugraheni et al., 2018; Takken and Rep, 2010; Zuriegat et al., 2021). In addition, proteolytic enzymes like aspartic protease FolASP, peptidase FolAPY1 and effector FolSvp2 have also been implicated in the infection process (Nishmitha et al., 2024; Wang et al., 2023; Li et al., 2024).

Although multiple effector proteins in *F. oxysporum* have been implicated in the infection process, few are individually sufficient to fully account for the pathogen’s virulence. This suggests the potential existence of additional pathogenic effector within *F. oxysporum* and indicates that the underlying mechanism of pathogenesis remains largely elusive. Previous transcriptomic analysis of *Foc* (Accession Numbers: SRR6793782–6793799) during infection of cucumber roots revealed significant up-regulation of a gene (*unigene758*encoding a putative secretory protein), designated FoCup (Huang X. Q. et al., 2019). To characterize its biological function, this study employed fungal genetic transformation to generate knockout and complementation mutants. The phenotypic effects of FoCup on vegetative growth and pathogenicity were systematically investigated.

## Materials and methods

### Fungal isolates and plant materials

Experiments were conducted with the following *Foc* isolates: (i) the weakly virulent strain Foc-3b (ACCC39326), isolated from cucumberin Langfang, and (ii) its virulence-enhanced variant, Ra-4, derived from Foc-3b via serial passages on a resistant cucumber cultivar in our previous study (Huang X. et al., 2019; Huang X. Q. et al., 2019). The *Foc*-susceptible cucumber cultivar “Zhongnong 6” used in this study was purchased from Zhongshu Seed Industry Technology Co., Ltd. (Beijing, China).

### RNA extraction and gene expression analysis

Cucumber seedlings with two cotyledons and one true leaf were transplanted in greenhouse and inoculated via root drenching with 5 mL of a *Foc* conidial suspension at 1 × 10^6^ conidia/mL. Root samples were collected at 6, 24 and 120 h post-inoculation (hpi) for further analysis. A mixture of conidia and mycelia harvested from *Foc* cultures grown in potato dextrose broth (PDB) served as control as 0-hpi control. Total RNA was extracted from the root samples and corresponding controls using RNeasy Plant Mini Kit (Qiagen, Duesseldorf, Germany). First-strand cDNA was subsequently synthesized from the extracted RNA using the cDNA FastQuant RT Kit (Tiangen, Beijing, China), strictly following the manufacturers’ protocols for both procedures. The transcription levels of *FoCup* in *Foc* were determined via quantitative reverse transcription polymerase chain reaction (qRT-PCR) on a Bio-Rad iQ 5 Real-Time System (Bio-Rad, California, United States). Reactions were performed using SYBR Premix Ex Taq (Takara, Dalian, China) with gene-specific primers qRT-FoCup-F/R (listed in Supplementary Table S1). The *EF1α* gene (GenBank accession number: KP274074) was employed as an internal reference for normalization. Relative gene expression was calculated using the 2^−ΔΔCT^ method as described by Livak and Schmittgen (2001). Three independent biological replicates were performed for each sample, and the entire experiment was repeated three times to ensure reproducibility.

### Bioinformatics characterization and phylogenetic analysis of FoCup

The *FoCup* (GenBank accession no. SUB14567058) copy number was determined by screening the *Foc* genome, while its DNA sequence was analyzed by aligning with reference genomes using BLAST (Basic Local Alignment Search Tool). Conserved domains in the protein FoCup were predicted using InterPro^1^ and SMART^2^. Signal peptide and transmembrane regions of the protein were analyzed with SignalP-5.0^3^ and TMHMM^4^, respectively. Homologs of FoCup were obtained through the BLAST program within UniProt^5^. A phylogenetic tree was constructed via the neighbor-joining method in MEGA software (version 11.0), with branch support assessed through 1,000 bootstrap replicates.

### Functional validation of FoCup signal peptide

Signal peptide sequences was initially predicted using SignalP-5.0. The encoding gene fragment was amplified from *Foc* genomic DNA via PCR with primers listed in Supplementary Table S1, and cloned into pSUC2 vector (Yin et al., 2018). The recombinant plasmid pSUC2-*FoCup* was transformed into yeast strain YTK12. Transformants were cultured on complete medium-tryptophan deficient (CMD-W) and yeast peptone raffinose adenine agar (YPRAA) medium, to assess invertase secretion-dependent growth. The pSUC2-*Avr1b* and empty pSUC2 vectors were used as positive and negative controls (Dou et al., 2008), respectively. For further confirmation of invertase secretion via enzymatic activity, transformed yeast strains were cultured in CMD-W liquid medium at 30 °C for 16 h. Cells were harvested, resuspended in a colorless 0.1% 2,3,5-triphenyltetrazolium chloride (TTC, Solarbio, Cat# T8170) solution, and incubated to observe the reduction of TTC to insoluble red triphenyl formazan (Oh et al., 2009).

### Subcellular localization of FoCup

The coding sequence of *FoCup*, without stop codon, was cloned into the binary vector pSuper1300 to generate a fusion with the green fluorescent protein (GFP) tag, resulting in the construct pSuper1300-FoCup-GFP. This plasmid was introduced into *Agrobacterium tumefaciens* strain GV3101 (Bartholomew et al., 2019). For plasma membrane localization, a marker construct expressing *AtPIP2A*-mCherry (CD3-1007) was co-expressed (Nelson et al., 2007). Transient expression in *Nicotiana benthamiana* leaves was performed as described by Wang et al. (2015). GFP and mCherry fluorescence were visualized using a Zeiss LSM 510 confocal laser-scanning microscope with a 40× objective lens at excitation wavelengths of 488 and 561 nm, respectively.

### Generation and validation of *FoCup* gene replacement and complementation mutants

To generate *FoCup* gene replacement mutants, a hygromycin resistance gene (*hph*) fragment was amplified from the plasmid pKH-KO with primers HphF/HphR. Upstream and downstream flanking sequences of *FoCup* were amplified using primer pairs of SP3-UF/UR and SP3-DF/DR, respectively. A recombinant fragment replacing *FoCup* with *hph* was assembled via double-joint PCR (Yu et al., 2004) with primers SP3-NF/NR. For genetic complementation, the *FoCup* coding sequence was amplified with primer CFLAG-FoCup-F/R and cloned into the XhoI-linearized pFL7 vector (Bruno et al., 2004; Zhou et al., 2011). The resulting complementation construct was transformed into the yeast strain XK1-25. Gene deletion fragments and complementary vectors were introduced into *Foc* WT (Ra-4) and Δ*FoCup* protoplasts, respectively. Protoplasts were prepared through enzymatic digestion of fungal cell walls (Turgeon et al., 1987). The transformants were screened on PDA plates supplemented with hygromycin B or Geneticin, and validated by PCR and DNA sequencing.

### Pathogenicity assay

Pathogenicity assays were conducted by inoculating cucumber seedlings grown in sterile substrate with 1 mL spore suspension at 1 × 10^6^ conidia/mL from WT strain (Ra-4) and knockout mutant Δ*FoCup*, complementation mutant Δ*FoCup*+*FoCup* cultured in PDB medium. Sterile water was used as a negative control. Each treatment composed of 20 seedlings. Disease severity was assessed at 14 and 21 days post-inoculation (dpi) by using the following grading system (Qian et al., 2022): 0, no symptoms; 1, mild symptoms (cotyledons wilting or slight wilting of some true leaves); 2, wilting of one true leaf wilt or severe cotyledon wilting; 3, three or more leaves exhibiting symptoms; 4, whole plant lodged but alive; 5, plant death. The disease index was calculated as follows: Disease index = [*Σ* (number of diseased plants × grade value)/(total plants × maximum grade value)] × 100. All experiments were performed with three independent biological replicates.

### Tests on mycelial growth, conidiation, and stress tolerance

The mycelial growth rates of WT strain (Ra-4), knockout mutant Δ*FoCup* and complementation mutant Δ*FoCup*+*FoCup* were tested by daily cross-measurement of colony diameters on PDA plates incubated at 28 °C. Conidial production was quantified by counting conidia in PDB cultures using a hemocytometer. The cultures were grown at 28 °C with agitation at 180 rpm. Then the germination rate was determined by diluting the spore suspension to 1 × 10^5^ conidia/mL. Then, 100 μL of the spore suspension was spread onto PDA medium plates, which were incubated upside down at 28 °C for 8 h. Then, conidial germination was observed under a microscope. Conidial germination was determining when the germ tube length exceeded half of the diameter of the original conidium. Fungal mycelial dry weight was determined by culturing the tested strains in PDB at 28 °C with 180 rpm for 5 d, harvesting from 20 mL of the liquid culture with filtration through three layers of sterilized miracloths, and drying the filtrates placed in a 9 cm petri dish within a hot-air oven at 70 °C for 12 h when constant weight was achieved. Stress response of fungal strains were assessed by culturing them on PDA plates supplemented with individual stress-inducing agents. Colony diameters were measured after 6 d of incubation at 28 °C. The tested agents included cell wall stressors: congo red (CR, 0.2 g/L), sodium dodecyl sulfate (SDS 0.2 g/L); osmotic stressors: glycerol (1 M), glucose (1 M), sorbitol (1 M), sodium chloride (NaCl, 1 M), or potassium chloride (KCl, 1 M). All the experiments were repeated three times.

### Statistical analyses

Statistical analyses were performed using either the Student’s *t*-test or one-way analysis of variance (ANOVA). Significant differences were defined by probability values at *p* < 0.05.

## Results

### Upregulation of FoCup in Foc during cucumber infection

**Figure 1:**
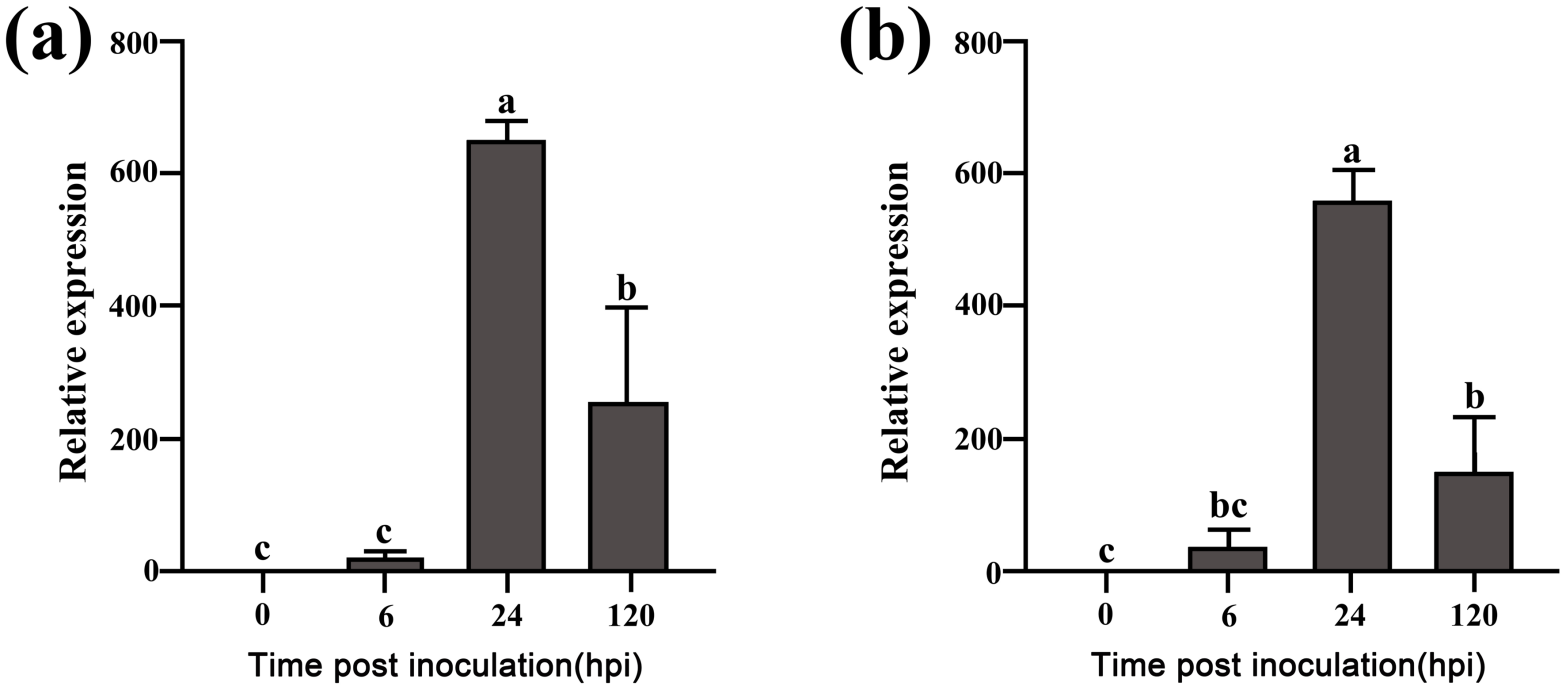
Relative expression of *FoCup* gene in *Foc* isolates Foc-3b **(a)** and Ra-4 **(b)** during their infection on cucumber plants at different hours post-inoculation. Expression levels were normalized to the 0 hpi control, which was the mixture of conidia and mycelium cultured in PDB at 28 °C. *EF1α* (elongation factor 1α) gene was used as internal reference gene for normalization. All data were calculated using the 2^−∆∆Ct^ method and represented the mean values derived from three independent biological replicates. Letters above the bars indicate statistically significant differences at *p* < 0.05.

Quantitative PCR analysis revealed a significant upregulation of *FoCup* expression in *Foc* during cucumber infection. At 24 hpi, transcript levels increased by over 323-fold in isolate Foc-3b (Figure 1a), and by more than 555-fold in isolate Ra-4 (Figure 1b). Elevated expression persisted until 120 hpi in both isolates, suggesting a close involvement of *FoCup* in *Foc* pathogenesis.

### Domain organization and phylogenetic analysis of the FoCup protein

**Figure 2:**
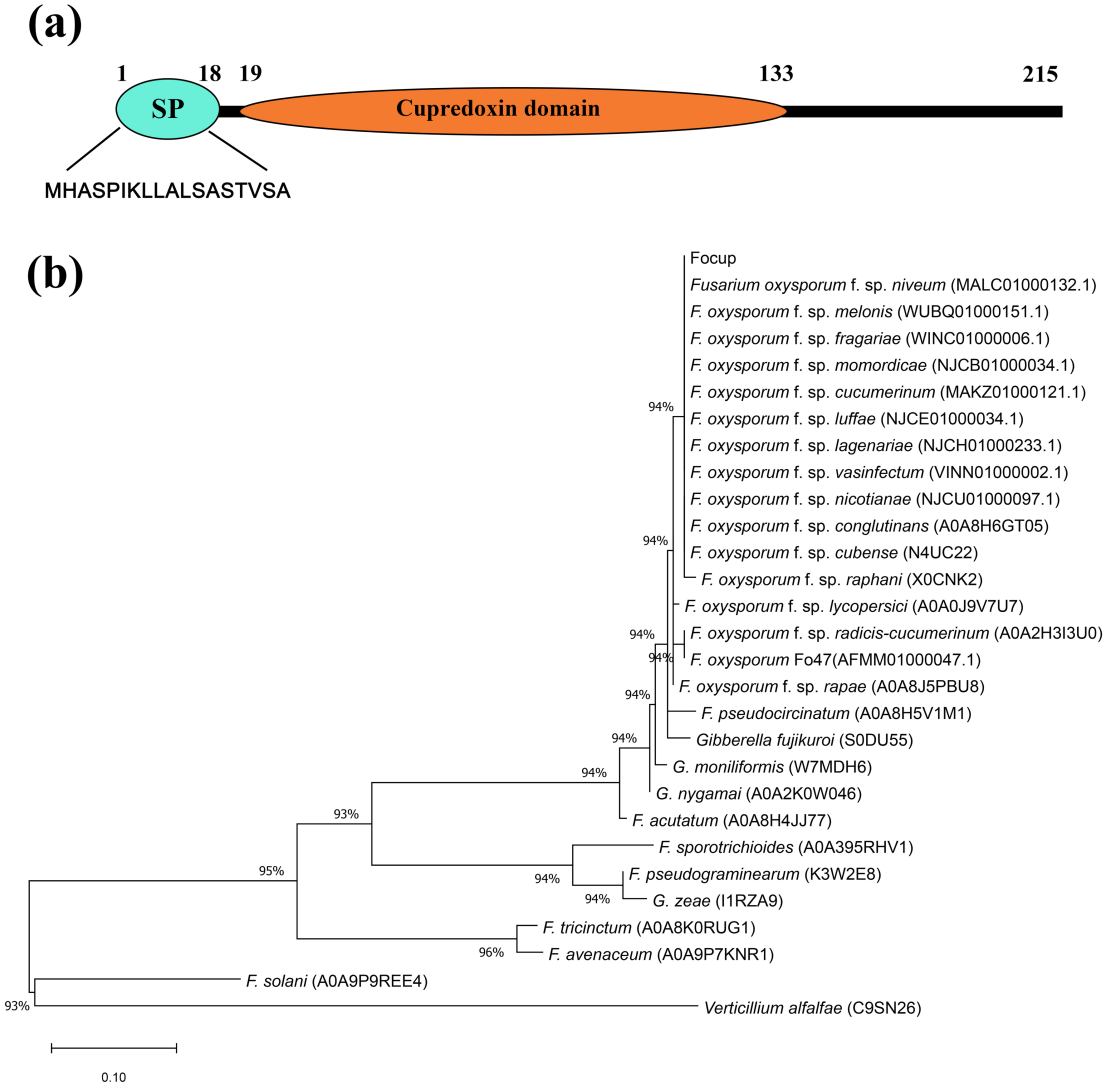
Schematic representation of FoCup protein domain architecture and phylogenetic analysis of FoCup with homologs from other fungi. **(a)** Signal peptide and domains of the FoCup protein. **(b)** Multiple sequences were aligned by using Clustal X, and the phylogenetic tree was constructed with MEGA 11.0 under the Neighbor-Joining method. UniProt accession numbers were shown in parentheses. Bootstrap values from 1,000 replicates are indicated at the nodes. Scale bar = 0.05, representing genetic distance.

As predicted by InterPro analysis, *FoCup* was a single copy gene mapped to chromosome 2 of *Forc* (CM008288.1) and *Fol* (NC_030987.1), a core chromosome. It encoded a protein containing a cupredoxin domain (Figure 2a), from which its name is derived. The gene has a total length of 696 bp, including a 51 bp intron and a 645 bp coding sequence (CDS). Bioinformatic prediction using SignalP-5.0 indicated the presence of an N-terminal secretory signal peptide spanning residues 1–18, with a cleavage site located between residues 18–19. Based on BLASTP analysis and phylogenetic tree construction using MEGA 11.0 (Figure 2b), the FoCup in *Foc* shared identical amino acid sequences with those in other *F. oxysporum* formae speciales, including those infecting cucurbitaceae plants, except for f. sp. *radicis-cucumerinum*. Among the 17 formae speciales analyzed, only four formae speciales, f. sp. *rapae*, f. sp. *raphani*, f. sp. *lycopersici* and the nonpathogenic strain *Fo47*, exhibited several amino acid variations (Supplementary Figure S1). Notably, the secretory signal peptide domain was highly conserved across *F. oxysporum* formae speciales. In contrast, the FoCup itself is not conserved in other *Fusarium* species (Figure 2b) or other non-*Fusarium* species (Supplementary Figure S2). Additionally, amino acid variations were observed within the cupredoxin domain and the C-terminal region among the orthologs.

### Validation of FoCup as a secreted protein in *Foc* and its subcellular localization

**Figure 3:**
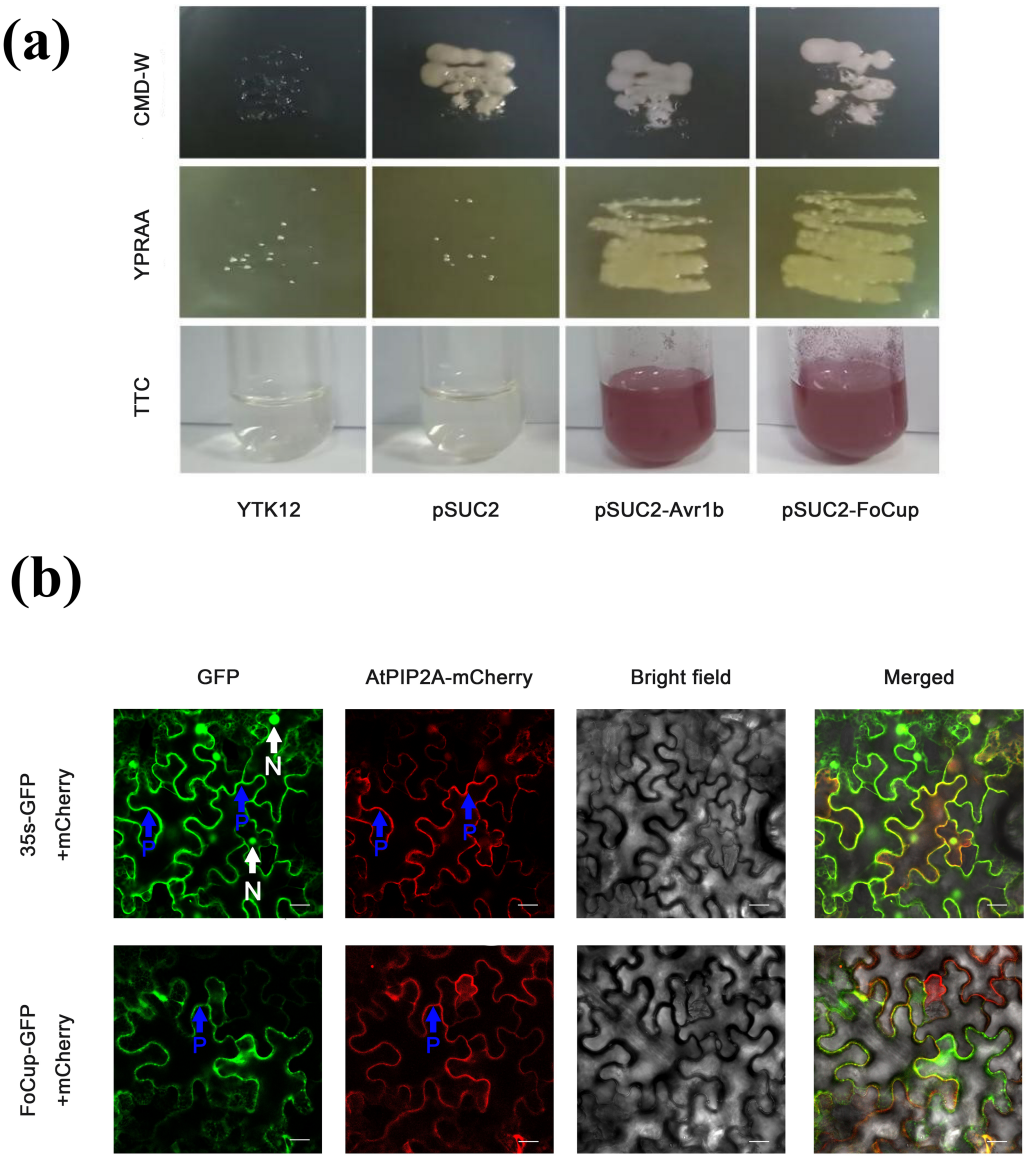
Functional validation of the signal peptide of FoCup and subcellular localization in *N. benthamiana* leaves. **(a)** Functional validation of FoCup signal peptide using a yeast secretion assay. Yeast transformants carrying the pSUC2-*FoCup* fusion were selected to grow on CMD-W (Trp-free) an YPRAA media for assessment of invertase secretion. YTK12 strains harboring empty pSUC2 or no vector served as negative controls. **(b)** Subcellular localization of FoCup in *N. benthamiana* leaves 36 h after *Agrobacterium-mediated* transformation. FoCup-GFP or AtPIP2A-mCherry was transiently expressed in *N. benthamiana* leaves and imaged 36 hpi. 35 s-GFP expressed alone was used as a nuclear localization control (green florescent protein), while the plasma membrane was labeled with mCherry (red fluorescent protein). Nucleus (N) are indicated with white arrows, and the plasma membrane (P) of plant cells are indicated with blue arrowheads. Bars = 20 μm.

Yeast strains expressing a *FoCup-Avr1b* fusion grew well on YPRAA medium (Figure 3a), indicating that both the signal peptides of *FoCup* and *Avr1b* enabled the YTK12 strain to secrete invertase. This enzyme hydrolyzed raffinose in the medium, providing a carbon source for growth. In addition, these strains reduced 2,3,5-triphenyltetrazolium chloride (TTC) to red triphenyl formazan. In contrast, the negative controls YTK12 and transformants carrying the empty pSUC2 vector showed no color change (Figure 3a). These results confirmed that FoCup functioned as a secretory protein.

Subcellular localization of FoCup with transient co-expression of the FoCup -GFP and the mCherry-tagged plasma membrane marker (AtPIP2A-mCherry) in *N. benthamiana* leaves demonstrated that *FoCup*-GFP localized specifically to the plasma membrane, including the cell periphery and apoplastic regions and co-localized with AtPIP2A-mCherry (Figure 3b). In contrast, GFP alone displayed diffuse localization throughout the cytoplasm and nucleus (Figure 3b).

### Effects of FoCup on pathogenicity of *Foc* in cucumber

**Figure 4:**
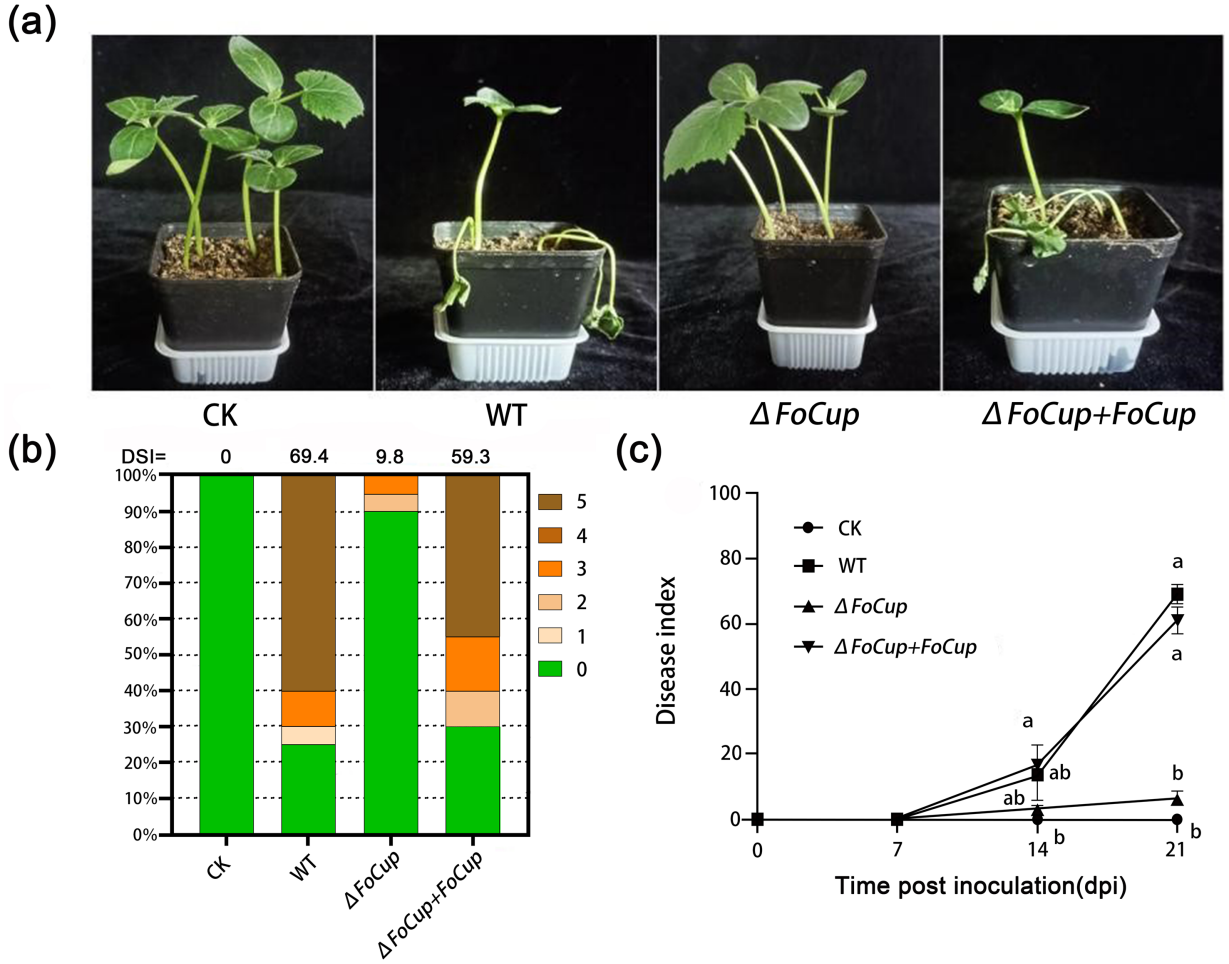
Involvement of FoCup in pathogenicity of *Foc* on cucumber. **(a)** Pot-cultured 20 seedlings in each treatment were inoculated by drenching conidial suspension at 1 × 10^6^ conidia/mL of WT strain (Ra-4), Δ*FoCup* and Δ*FoCup+FoCup* mutants, respectively. Sterile water was used as a negative control. Photos were taken 21 days post inoculation (dpi). Experiment was repeated three times. **(b)** Disease severity index (DSI) were recorded at 21 dpi. **(c)** Disease indices were calculated based on assessments at 7, 14, and 21 dpi using a 0–5 rating scale (0: healthy; 5: plant death). Letters above the bars indicate statistically significant differences at *p* < 0.05.

Pathogenicity tests revealed that cucumber seedlings inoculated with the WT strain (Ra-4) and the knockout mutant Δ*FoCup*, the complemented mutant Δ*FoCup*+*FoCup* showed typical wilting symptoms 3 weeks post-inoculation (wpi), whereas plants inoculated with the knockout Δ*FoCup* mutant exhibited no obvious symptoms (Figure 4a). Disease assessment confirmed that deletion of *FoCup* significantly reduced disease incidence (Figure 4b), and decreased the disease severity index by 62.5 and 54.5 compared to the WT strain (Ra-4) and the complemented mutant (*ΔFoCup+FoCup*), respectively (Figure 4c). These data demonstrated that *FoCup* plays a critical role in virulence of *Foc*.

### Effects of *FoCup* on conidial germination, mycelial growth and sporulation in *Foc*

**Figure 5:**
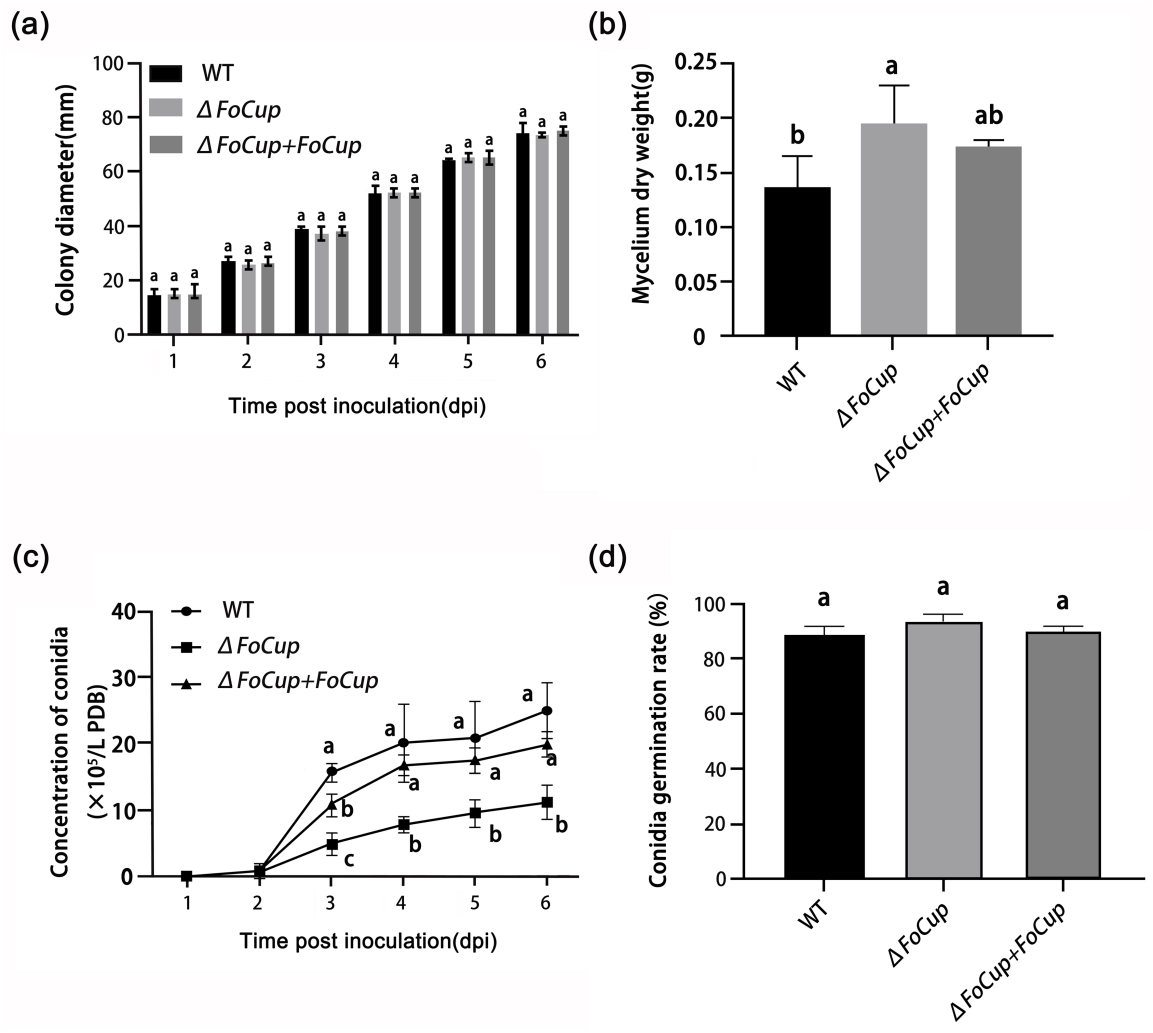
Effects of *FoCup* deletion on mycelium growth, conidia production, and germination of *Foc*. **(a)** Mycelial growth rates as indicated by daily cross-measured diameters of WT (Ra-4), Δ*FoCup* and Δ*FoCup+FoCup* colonies cultured in PDA plates at 28 °C. **(b)** Mycelial dry weight was determined by culturing the tested strains in PDB at 26 °C with 180 rpm for 5 d, harvesting from 20 mL of the liquid culture with Miracloth filtration, and drying in a 9 cm petri dish within a hot-air oven at 70 °C for 12 h when constant weight was achieved. **(c)** Conidiation was quantified by inoculating each strains into 100 mL PDB medium and culturing at 28 °C with 180 rpm. Conidia in the culture broth were counted daily using a hemacytometer to determine the conidial production. **(d)** Conidia germination rate was determined by diluting the spore suspension to 1 × 10^5^ conidia/mL. Then, 100 μL of the spore suspension was spread onto PDA medium, which were incubated upside down at 28 °C for 8 h. Then, conidial germination was observed under a microscop. For each plate, 100 conidia were randomly observed, and the germination rate was calculated based on these observations. Letters above bars indicate statistically significant differences at *p* < 0.05 as determined by one-way ANOVA with Tukey’s multiple-comparison test.

Although no significant differences in colony diameter were observed among the WT strain (Ra-4), the knockout mutant Δ*FoCup* and the complemented mutant Δ*FoCup*+*FoCup* after 6 d of culture on PDA (Figure 5a), assays in PDB revealed that deletion of *FoCup* significantly increased the mycelial dry weight (Figure 5b) and reduced sporulation (Figure 5c). However, knockout of *FoCup* had no effect on spore germination (Figure 5d).

### Effects of *FoCup* on stress tolerance of *Foc*

**Figure 6:**
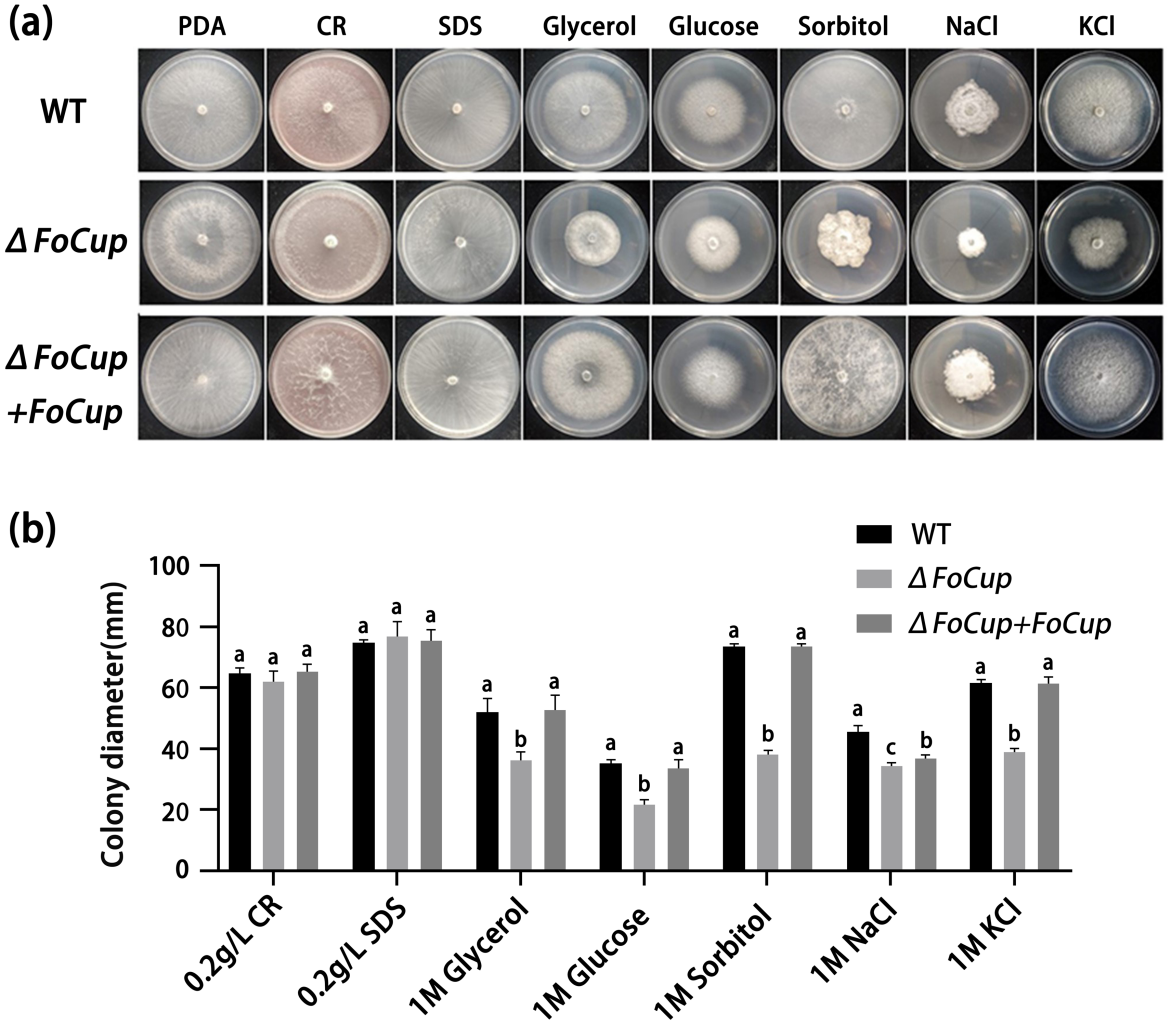
Effects of *FoCup* on stress tolerance of *FOC* as indicated by colony morphology **(a)** and colony diameter **(b)** of WT strain (Ra-4) and Δ*FoCup*, (Δ*FoCup*+*FoCup*) mutants cultured at 28 °C for 6 d in PDA plates amended with 0.2 g/L Congo red (CR), 0.2 g/L sodium dodecyl sulfate (SDS), 1 M glycerol, 1 M glucose, 1 M sorbitol, 1 M NaCl, and 1 M KCl, respectively. Letters above the bars indicate statistically significant differences at *p* < 0.05.

Evaluation of the *FoCup* role in stress tolerance of *Foc* showed that there was no significant difference in growth rates of WT strain (Ra-4) and knockout mutant Δ*FoCup*, complemented mutant Δ*FoCup+FoCup* mutants in PDA plates amended with cell wall stressors 0.2 g/L CR and 0.2 g/L SDS. However, Δ*FoCup* mutant exhibited markedly reduced growth on the media amended with glycerol, sorbitol, sorbitol, NaCl and KCl in comparison with those of WT strain (Ra-4) and complemented mutant Δ*FoCup+FoCup* mutant strain (Figure 6), indicating that *FoCup* contributes to osmotic stress tolerance in *Foc*.

## Discussion

In this study, we identified and characterized a novel secreted protein, designated FoCup, which contains a cupredoxin domain in *F. oxysporum*. This was the first reported secreted protein with a cupredoxin domain in *Fusarium*. FoCup shows high sequence conservation within the *Fusarium* genus, yet its biological function has remained unexplored. Proteins with high homology to FoCup have not been functionally characterized, indicating that FoCup represents to a new class of secreted proteins in *Fusarium*. Furthermore, we demonstrated that FoCup deeply involved in the pathogenesis of *Foc* on cucumber plants.

Secreted proteins can determine pathogenesis by affecting one of the multiple infection steps, including recognition, adhesion and penetration etc. for the pathogen to make host plants diseased successfully. *SIX1* was the first reported effector in *F. oxysporum*, identified in *Fol* in 2004 (Rep et al., 2004). To date, 14 xylem-secreted proteins have successively identified in *Fol* and designated *SIX1* through *SIX14* in *F. oxysporum* (Houterman et al., 2007; Schmidt et al., 2013). The *SIX1*-*SIX6* genes have been confirmed to be specifically expressed in *Fol* only after it invades the host roots, and their expression levels gradually increase with the duration of host infection, generally reaching a peak at 5–7 dpi (Gawehns et al., 2014). Beyond *Fol*, nine *SIXs* homologs in *F. oxysporum* f. sp. *cubense* exhibited distinct temporal expression patterns during infection. Whereas *SIX2*, *SIX4*, and *SIX6* showed minimal induction (1–1.5 fold) at 24 hpi, the majority peaked later (5–7 dpi) with marked induction. Among these, *SIX9* was upregulated 35-fold, and others by at least 5-fold. Moreover, the *SIX8* gene (89% homologous to *FolSIX8*) exhibited progressive upregulation, reaching an 8-fold increase by 7 dpi (An et al., 2019). Collectively, the SIX proteins are the most well-characterized effectors in *F. oxysporum*. These vascular-targeted, accessory chromosome-encoded proteins exhibit induced expression during host infection.

In addition to the known SIX effectors, several novel secreted proteins were identified. Despite not being exclusively located on accessory chromosomes, these genes were highly upregulated at an earlier time point during host infection, and are likely involved in pathogenicity. For instance, *FolSvp1* and *FolSvp2*, located on core chromosomes in *Fol*, show barely detectable expression *in vitro*, they are strongly induced upon contact with tomato roots, resulting in significant upregulation of both their transcripts and secreted protein levels (Li et al., 2022, 2024). The F*olAsp* gene, located on a core chromosome of *Fol* and coding a secreted aspartic protease, was highly induced in the tomato apoplast (Wang et al., 2023). Its expression peaked at 9 hpi, with levels exceeding those under in vitro conditions by more than 150-fold. While expression slightly declined thereafter, it remained elevated (Wang et al., 2023). In addition to individual proteins mentioned above, lysine acetylome profiling of the *Fol* secretome identified 26 core chromosome-encoded secreted proteins that were induced during infection, including 10 that showed a marked upregulation which peaked at 24 hpi (Li et al., 2020). Similarly, the *Fosp9* gene, located on an accessory chromosome of *F. oxysporum* f. sp. *cubense* race 4 and coding a secreted protein, was massively upregulated during host infection. Despite its negligible expression in vitro, transcript levels increased by more than 400-fold upon inoculation, irrespective of whether the host cultivar was resistant or a susceptible (Guo et al., 2022). Beyond *F. oxysporum*, the *SsCP1* gene in *Sclerotinia sclerotiorum* was strongly induced during infection, with expression increasing by more than 8-fold at 12 hpi, contrasting with its negligible in vitro levels (Yang et al., 2017). In this study, we found that *FoCup* gene, localized on the core chromosome, was barely expressed in vitro, but was dramatically up-regulated at 24 hpi. Although its expression level decreases by half at 120 hpi, it remains highly expressed. Based on its minimal expression during the saprophytic phase but dramatic up-regulation during the parasitic phase, we hypothesize that this gene plays a critical role in host infection and pathogenesis.

This study reports the initial identification of a secreted cupredoxin-domain-containing protein in *Fusarium*, whose high-homology homologs have not been functionally characterized to date. The sequence of N-terminal secretory signal peptide region in FoCup is completely conserved across all formae speciales. However, there is some divergence within the cupredoxin domain, suggesting that while the exocytosis pathway of this protein remains the same among different formae speciales, the interacting proteins after entering the host may differ. The phylogenetic distance of FoCup was substantial compared to its homologs in other *Fusarium* species, indicating significant functional divergence among these species. Proteins containing a cupredoxin domain have been reported to be involved in various biological processes in fungi and plants. MoPtep1, an effector protein containing a cupredoxin domain secreted by *Magnaporthe oryzae*, was localized in rice plant peroxisomes and demonstrated to participate in peroxisomal redox reactions by binding copper ions, and thereby regulating intracellular redox balance and influencing pathogen virulence (Ning et al., 2022). ZmSKS13, a cupredoxin domain-containing protein in maize, plays a critical role in kernel development via modulation of redox homeostasis (Zhang et al., 2021). In soybean, the upregulation of cupredoxin family genes in response to soybean cyst nematode infection suggests they may function in plant disease defense via phytohormone signaling pathways (Zhang et al., 2022). Further investigation into the functional role of FoCup in *F. oxysporum* will be of great significance for advancing our knowledge about proteins with the cupredoxin domain.

Sporulation is critically important for a pathogenic fungus, as it is essential for both dissemination and infection (Gow et al., 2002; Uddin, 2023). However, many secreted proteins are only expressed under host-induced conditions to affect virulence, without influencing mycelial growth and conidiation. A common feature among effector protein encoding genes in *Fo* like *FolAsp* (located on a core chromosome), *FolSCP1*/*SIXs* (accessory chromosome) in *Fol*, and *Fosp9* (core chromosome) in *F. oxysporum* f. sp. *cubense* is that they are required for full virulence, while being dispensable for normal growth *in vitro* (Wang et al., 2023; Qian et al., 2025; Guo et al., 2022). These findings indicate that these effector genes, regardless of their location on accessory or core chromosomes, do not affect in vitro conidiation. However, some secreted proteins involved in both virulence and fungal growth. For instance, knockout of *F. oxysporum* f. sp. *cubense* race 4 FoSP1 reduces conidiation, spore germination, and pathogenicity (Wang et al., 2022). Beyond the genus *Fusarium*, while deletion of the biotrophy-associated secreted protein BAS2 impairs conidiation in *Colletotrichum gloeosporioides* (An et al., 2018), overexpression of the apoplastic effector BAS4 in *Magnaporthe oryzae* enhances spore production and stress tolerance, facilitating pathogen colonization and dissemination (Wang C. et al., 2019). In this study, we demonstrated that knockout of the *FoCup* not only impaired virulence severely, but also significantly reduced sporulation and enhanced mycelial dry weight, indicating that *FoCup* regulates the growth morphology of *Foc* by shifting it from mycelial growth to sporulation when deficient. This suggests that the observed reduction in conidial production may underlie the diminished virulence. However, the specific role of this gene during conidiogenesis warrants further investigation.

Fungi respond in various ways to exogenous stresses in order to maintain cell shape and normal physiological processes (Leng and Zhong, 2015; Wang C. et al., 2019). *F. oxysporum* maintains cellular morphology and function by regulating intracellular osmolarity under hyperosmotic conditions (Shoaib et al., 2018). Our results showed that deletion of the *FoCup* significantly increased the osmotic sensitivity of *Foc* to glycerol, sorbitol, NaCl, and KCl. This phenotype is attributed to cellular dehydration and subsequent growth inhibition caused by a defect in osmoregulation. However, the cupredoxin domain-containing MoPtep1 protein is specifically required for the pathogenicity of *M. oryzae* but is dispensable for its responses to diverse stresses, including oxidative, osmotic, cell wall, and metal cation challenges (Ning et al., 2022). Altered sensitivity to osmotic stress will definitely affects the pathogen’s colonization and survival in soil and host plants (He et al., 2018; Wang et al., 2018), echoing the finding mentioned above that the FoCup protein was involved in pathogen’s early infection stage in cucumber plants. The role of gene *FoCup* in osmotic sensitivity, and its consequent impact on conidiation and pathogenicity, merits further investigation.

In conclusion, this study identified a novel effector gene in *F. oxysporum*, FoCup, containing a cupredoxin domain. Although located on a core chromosome, FoCup influences both conidiation *in vitro* and virulence in planta. Consequently, its encoded protein FoCup represents a promising target for chemical control and therefore warrants further investigation to elucidate its precise mechanisms in sporulation and pathogenesis.

## Data Availability

The datasets presented in this study can be found in online repositories. The names of the repository/repositories and accession number(s) can be found in the article/Supplementary material.

## Footnotes

1
http://www.ebi.ac.uk/interpro/

2
http://smart.embl-heidelberg.de/

3 https://services.healthtech.dtu.dk/services/SignalP-5.0/.

4
http://www.cbs.dtu.dk/services

5
https://www.uniprot.org/blast

## Supplementary material

SUPPLEMENTARY FIGURE S1 Sequence alignment was performed using ESPript3.0 for the FoCup and its homologs in different formae speciales of F. oxysporum. Fully conserved amino acid residues are marked with a red background, and divergent sites are shown in white letters.

SUPPLEMENTARY FIGURE S2 Phylogenetic tree of FoCup homologs across diverse taxonomic groups. The tree was constructed using the Neighbor-Joining method in MEGA 11.0 based on a Clustal X alignment. Numbers at nodes represent bootstrap values from 1,000 replicates. The scale bar corresponds to 0.10 genetic distance.
