# Supplementary figures and images for: FoCup, a secreted protein, is essential for virulence of Fusarium oxysporum f. sp. cucumerinum on cucumber
Source: Front Microbiol. 2025 Dec 17;16:1728884. doi: 10.3389/fmicb.2025.1728884 (PMC12753967; doi:10.3389/fmicb.2025.1728884)

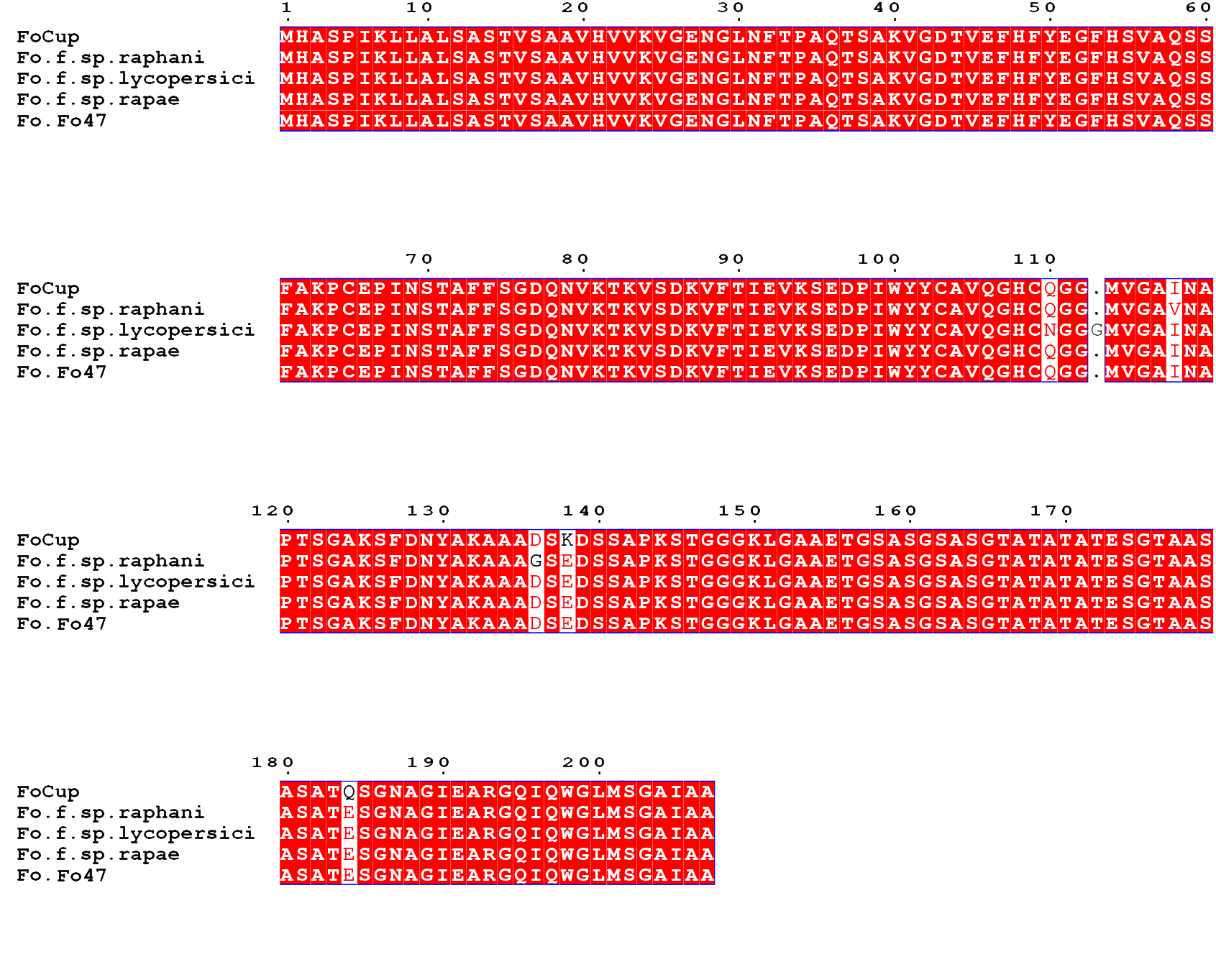

Supplement: SUPPLEMENTARY FIGURE S1 — Sequence alignment was performed using ESPript3.0 for the FoCup and its homologs in different formae speciales of F. oxysporum. Fully conserved amino acid residues are marked with a red background, and divergent sites are shown in white letters. [file Image_1.JPEG]

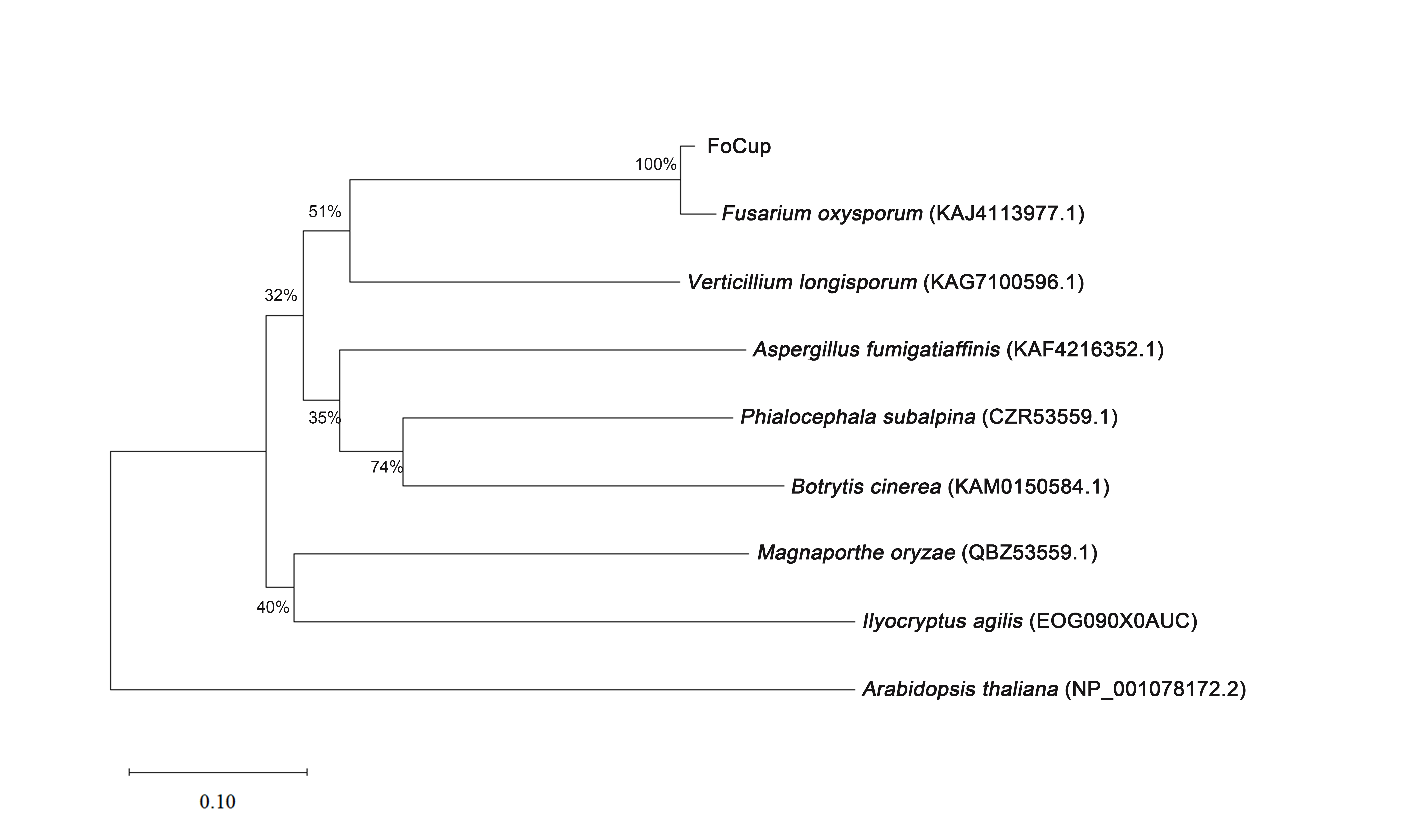

Supplement: SUPPLEMENTARY FIGURE S2 — Phylogenetic tree of FoCup homologs across diverse taxonomic groups. The tree was constructed using the Neighbor-Joining method in MEGA 11.0 based on a Clustal X alignment. Numbers at nodes represent bootstrap values from 1,000 replicates. The scale bar corresponds to 0.10 genetic distance. [file Image_2.JPEG]
