# Supplementary material for: FoCup, a secreted protein, is essential for virulence of Fusarium oxysporum f. sp. cucumerinum on cucumber
Source: Front Microbiol. 2025 Dec 17;16:1728884. doi: 10.3389/fmicb.2025.1728884 (PMC12753967; doi:10.3389/fmicb.2025.1728884)
Supplement: Supplementary file 3 [file Table_1.DOCX]

# Supplementary

**Table** S**1** **|** Primers used in this study.

| Primer  No | Primer | | Primer (5′-3′) | Purpose |
| --- | --- | --- | --- | --- |
| 1 | qRT-FoCup-F | AAGACCAAGGTCAGCGATAAG | | qRT-PCR primer of *FoCup* |
| 2 | qRT-FoCup-R | CGCTGGTAGGAGCGTTAATAG | |  |
| 3 | qRT-EF-F | CATCGGCCACGTCGACTCT | | qRT-PCR primer of *EF1* |
| 4 | qRT-EF-R | AGAACCCAGGCGTACTTGAA | |  |
| 5 | 1300-FoCup -BAMHI-F | gagctcggtacccggggatccATGCACGCCTCTCCCATCAAG | | Construction of 1300-FoCup-GFP vector |
| 6 | 1300-FoCup -SALI-R | gcccttgctcaccatgtcgacCATCATCAAACCGCCAAAGAGGG | |  |
| 7 | psuc2-FoCupF | cggaattttaattaagaattcATGCACGCCTCTCCCATCAAG | | Construction of pSUC2-FoCup vector |
| 8 | psuc2-FoCupR | cactatagggagaacctcgagCATCATCAAACCGCCAAAGAGGG | |  |
| 9 | psuc-avrb1F | cggaattttaattaagaattcATGCGTCTATCTTTTGTGCTT | | Construction of pSUC2-avrb1 vector |
| 10 | psuc-avrb1R | cactatagggagaacctcgagGCTCTGATACAGGTGAAAGGTGT | |  |
| 11 | pSUC2F | GGTGTGAAGTGGACCAAAGGTCTA | | Verifying the construction of pSUC2 vector |
| 12 | pSUC2R | CCTCGTCATTGTTCTCGTTCCCTT | |  |
| 13 | SP3-UF | TGCTCTACGATGACAAGCGATG | | PCR amplification of the upstream fragment of FoCup |
| 14 | SP3-UR | caaaataggcattgatgtgttgacctccTTTGGTGGTGGTGATAGGTGG | |  |
| 15 | SP3-DF | ctcgtccgagggcaaaggaatagagtaAGAATCTCCTGCCAGTCAACG | | PCR amplification of the downstream fragment of FoCup |
| 16 | SP3-DR | ACGATCACATACACTATTGCATTGAG | |  |
| 17 | SP3-NF | TAGCTTCCACATCGAGTGTTTATG | | PCR primers for identification of FoCup deletion transformants |
| 18 | SP3-NR | CAACGATATGCTCCTTGATCTCTG | |  |
| 19 | HphF | GGAGGTCAACACATCAATGCCTATTTTG | | PCR amplification of the hygromycin resistance gene |
| 20 | HphR | TACTCTATTCCTTTGCCCTCGGACGAG | |  |
| 21 | YZFoCup-F | ATGCACGCCTCTCCCATCAA | | Identification of FoCup deletion transformants |
| 22 | YZFoCup-R | CTACATCATCAAACCGCCAA | |  |
| 23 | CFLAG-FoCup-F | cagatcttggctttcgtaggaacccaatcttcaATGCACGCCTCTCCCATCAAG | | Construction of complement vector pFL7-FoCup |
| 24 | CFLAG-FoCup-R | ctttataatcaccgtcatggtctttgtagtcCATCATCAAACCGCCAAAGAGGG | |  |
| 25 | Pfl-Gene-F | CAGTCTCCCTTGCGGTTAAA | | Verifying the construction of pFL7-FoCup vector |
| 26 | Pfl-Gene-R | GGTATTGCCTTTGGCACTTTAC | |  |
